# 4D flow streamline characteristics of the great arteries twenty years after Lecompte and direct spiral arterial switch operation (DSASO) in simple TGA

**DOI:** 10.21542/gcsp.2016.29

**Published:** 2016-09-30

**Authors:** Hans-Hinrich Sievers, Léon M. Putman, Arash Kheradvar, Dominik Gabbert, Philip Wegner, Jens Scheewe, Mona Salehi-Ravesh, Hans-Heiner Kramer, Carsten Rickers

**Affiliations:** 1 Department of Cardiac and Thoracic Vascular Surgery, University Hospital of Schleswig-Holstein, Campus Luebeck, Ratzeburger Allee 160, 23538 Luebeck, Germany; 2 University of California Irvine, Edwards Lifesciences Center of Advanced Cardiovascular Technology, Irvine, CA 92697; 3 Department of Congenital Heart Disease and Pediatric Cardiology, University Hospital of Schleswig-Holstein, Campus Kiel, Arnold-Heller-Str. 3, 24105 Kiel, Germany

## Abstract

Transposition of the great arteries (TGA) is caused by discordance between the great arteries and the ventricles. If left untreated, this anomaly has a disastrous perspective. More recent surgical approach for correction includes the Lecompte technique in which the pulmonary bifurcation is transposed anterior to the aorta, which may be less physiologic. Although the early results are excellent, there is potential for future problems involving the great arteries and semilunar valves^1^. These potential problems necessitate the development of other improved surgical techniques^2^. Here we report an MRI 4D flow study related to a case of simple TGA whose primary surgical correction – direct spiral arterial switch operation (DSASO) – was performed twenty years ago in an attempt to restore physiologic arrangement among the great arteries and semilunar valves.

## Background

Simple TGA accounts for 5–7% of patients with congenital heart malformations and, untreated, has a bad prognosis with nearly 90% cases leading to death within one year. After a breakthrough of surgery for simple TGA inaugurating the two-stage arterial switch operation (ASO) by Yacoub in 1977^3^, the door was opened for successful anatomical correction.

**Figure 1.**
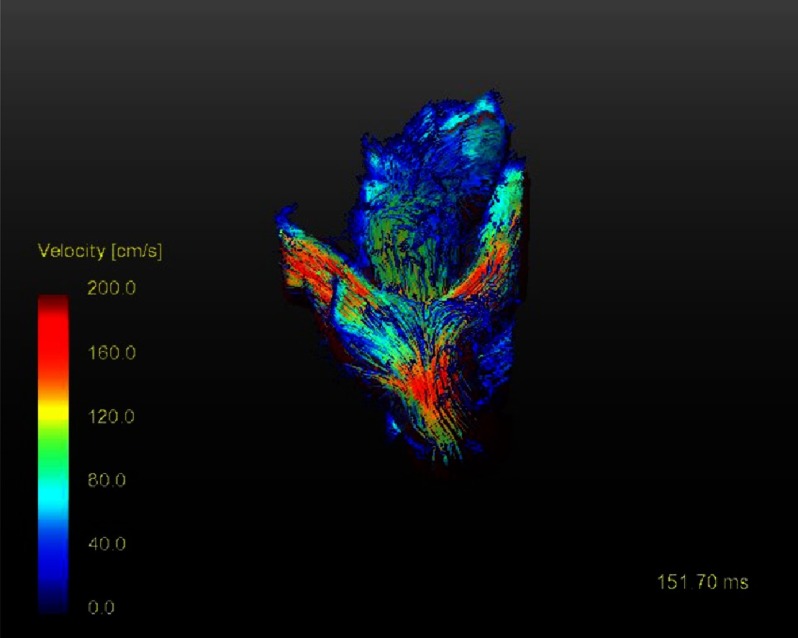
Blood streamlines twenty years after the Lecompte technique showing the flow acceleration particularly at the pulmonary bifurcation.

In the early 1980s, the heart-lung machine technology was refined to allow for new-borns to benefit from primary ASO. In 1981 the Lecompte technique^4^ was developed and used routinely to the present day. This technique transposes the pulmonary bifurcation in front of the aorta, which does not warrant spiral physiological blood flow in the great arteries (Figure 1). In outgrown patients some shortcomings of this technique have surfaced^1^, calling for new techniques^2^. In the early 1990s we performed a consecutive series of six patients performing a DSASO (for details see reference^5^) and re-evaluated these patients twenty years after the operation by MRI technique (for technical details see reference^5^) showing promising results^5^.

**Figure 2.**
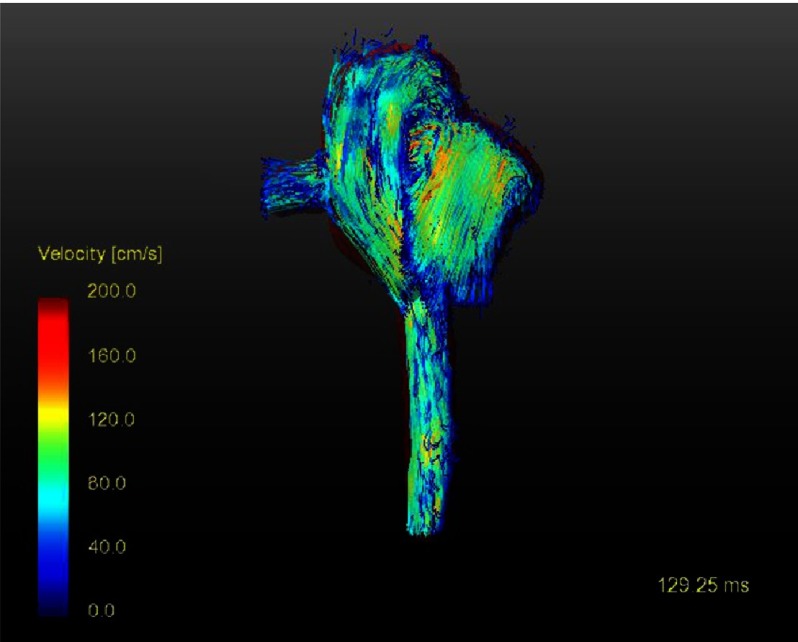
Blood streamlines twenty years after direct spiral arterial switch operation (DSASO).

**Figure 3.**
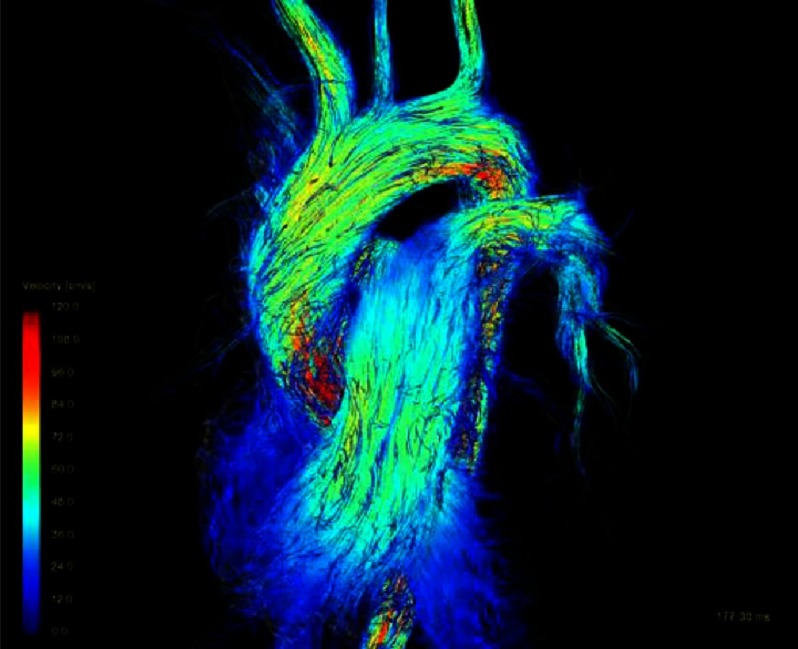
Blood streamlines in a healthy volunteer.

One example is presented here. The blood flow streamlines in the great arteries (Figure 2) show the spiral configuration comparable to a normal person (male; 33.4 years, no cardiac anomaly by echocardiography) (Figure 3) and more physiological than the streamlines in the Lecompte technique (Figure 1) with the blood flow vectors not in a spiral arrangement. There was no semilunar valve dysfunction in the DSASO patient. At the operation the TGA patient was 4 days old and had simple TGA with the aortic root 31°to the right of the pulmonary root. Figure 2 shows also that twenty years after DSASO the aortic or neo-pulmonary root has rotated somewhat to left indicating a potential morphogenetic adaptation to alter flow conditions during early age.

## Discussion

Normal anatomy is always the optimal solution in nature warranting normal physiology. This holds also true for surgery in congenital cardiac diseases. TGA is characterized by aorto-ventricular discordance. Theoretically re-transposing the great arteries would be the optimal solution. The near normal blood streamlines of the great arteries twenty years after DSASO provide some evidence that this re-transposition of the great arteries might be possible with excellent results. These data may stimulate a re-thinking on the optimal surgical technique of simple TGA preferably in cases with less rotation of the aortic root to the right of the pulmonary root. Refined operative techniques such as deliberately dissecting the arch and pulmonary arteries till the hilum to get more length of the great arteries as well as the transfer of the left coronary orifice as deep and posterior as possible in the related sinus of the pulmonary root including special anastomotic techniques like the trap door may be of advantage to prevent potential left coronary artery distortion by the rotation process of the neo-pulmonary root. In some cases an elongation of the pulmonary artery with a strip of autologous pericardium may allow for tension and torsion free anastomosis^6,7^.

### Lessons Learned

This report suggests that recreating ‘normal’ anatomical relationship during surgical treatment of complex congenital heart disease could have important functional implications. Furthermore, post-operative studies of 4D blood flow could provide important insights in planning future operations.
